# Remodelling of the hepatic epigenetic landscape of glucose-intolerant rainbow trout (*Oncorhynchus mykiss*) by nutritional status and dietary carbohydrates

**DOI:** 10.1038/srep32187

**Published:** 2016-08-26

**Authors:** Lucie Marandel, Olivier Lepais, Eva Arbenoits, Vincent Véron, Karine Dias, Marie Zion, Stéphane Panserat

**Affiliations:** 1 INRA, Univ Pau & Pays Adour, UMR 1419, Nutrition, Metabolism and Aquaculture, Saint Pée sur Nivelle, F-64310, France; 2 INRA, UMR 1224, Ecologie Comportementale et Biologie des Populations de Poissons, Saint Pée sur Nivelle, F-64310, France; 3 Univ Pau & Pays Adour, UMR 1224, Ecologie Comportementale et Biologie des Populations de Poissons, UFR Sciences et Techniques de la Côte Basque, Anglet, F-64600, France, Anglet, F-64600, France

## Abstract

The rainbow trout, a carnivorous fish, displays a ‘glucose-intolerant’ phenotype revealed by persistent hyperglycaemia when fed a high carbohydrate diet (HighCHO). Epigenetics refers to heritable changes in gene activity and is closely related to environmental changes and thus to metabolism adjustments governed by nutrition. In this study we first assessed in the trout liver whether and how nutritional status affects global epigenome modifications by targeting DNA methylation and histone marks previously reported to be affected in metabolic diseases. We then examined whether dietary carbohydrates could affect the epigenetic landscape of duplicated gluconeogenic genes previously reported to display changes in mRNA levels in trout fed a high carbohydrate diet. We specifically highlighted global hypomethylation of DNA and hypoacetylation of H3K9 in trout fed a HighCHO diet, a well-described phenotype in diabetes. *g6pcb2* ohnologs were also hypomethylated at specific CpG sites in these animals according to their up-regulation. Our findings demonstrated that the hepatic epigenetic landscape can be affected by both nutritional status and dietary carbohydrates in trout. The mechanism underlying the setting up of these epigenetic modifications has now to be explored in order to improve understanding of its impact on the glucose intolerant phenotype in carnivorous teleosts.

The rainbow trout (*Oncorhynchus mykiss*) is considered to be a glucose-intolerant species, displaying persistent hyperglycaemia after intake of a high-carbohydrate diet^1^,^2^,^3^. The biology of this strictly carnivorous fish has been thoroughly studied at physiological, biochemical and transcriptional levels since the 90’s to try to explain the mechanisms underlying their poor ability to use dietary carbohydrates^4^,^5^,^6^,^7^. The sequencing of the trout genome^8^ has recently opened new perspectives to improve the understanding of the phenotype observed by providing the possibility to consider the complexity of the genome and the fate of duplicated genes after the salmonid-specific fourth whole-genome duplication event (Ss4R). Using this new tool, Marandel *et al.*^9^ demonstrated that dietary carbohydrates in the trout can differentially affect expression of duplicated genes involved in gluconeogenesis, a pathway hypothesized not to be inhibited by dietary carbohydrates in carnivorous fish. For instance, five duplicated genes encoding glucose-6-phosphatase (G6pc), the enzyme catalyzing the last stage of gluconeogenesis, were identified and found to be differentially regulated by dietary carbohydrates *in vivo*^9^,^10^ and also by insulin, glucose and amino acids *in vitro*^11^. The atypical up-regulation of two of these five genes (*g6pcb2* ohnologs; ohnologs are paralogs formed by a whole genome duplication event) by dietary carbohydrates or insulin *in vitro* was hypothesized to be involved in the establishment of the glucose-intolerant phenotype in trout fed a high carbohydrate diet.

The duplicated genes that are differentially expressed in trout in response to a diet rich in carbohydrate share high level of sequence homology^9^. How these genes are differentially regulated remains however to be elucidated.

Epigenetic modifications are possible mechanisms through which nutritional status could initiate a metabolic memory by changing the chromatin structure and consequently the regulation of the genes, and hence their transcription^12^. Indeed changes in the epigenome are partly in response to environmental factors, including nutritional status, which will subsequently lead to adjustment of metabolism. This will insure that the cells respond promptly to environmental stimuli^13^,^14^,^15^. Modulation of epigenetic marks at the target gene loci as well as at the global level related to dietary carbohydrates have been thoroughly described in mammals, mainly linked to metabolic diseases, such as diabetes and hyperglycaemia, one of the most important feature of diabetes. Most of these investigations have highlighted global epigenetic changes in diabetic animals such as DNA hypomethylation and modification of histone marks such as hypoacetylation of H3K9, and hypermethylation of H3K4 and H3K9^16^,^17^,^18^, a phenotype also observed under hyperglycaemic conditions in mammals^19^,^20^ and in zebrafish^21^. Several studies carried out to explain the nutritional programming of metabolic diseases have also reported epigenetic modifications at gluconeogenic gene loci in mammals. For instance, in male piglets exposed *in utero* to a low protein diet, the *G6pc* promoter in the liver displayed a hypomethylation of DNA and an increase in H3K4me3 and H3K9me3 compared to controls^22^. Similarly, mice pups born of mothers fed a high fat diet during pregnancy displayed several histone modifications along the *Pck1* promoter^23^. A diabetic environment has also been shown to induce epigenetic changes at gluconeogenic gene loci^24^,^25^.

There is little information concerning epigenetic modifications at global and target gene levels mediated by nutritional status or by dietary carbohydrates and their physiological consequence (*i.e.*, hyperglycaemia) in rainbow trout. Indeed, only one study has reported global DNA hypomethylation in the livers of trout fed a high carbohydrate diet without prospecting for histone mark changes at glucose metabolism-related gene loci^26^. In the present study we therefore first assessed in trout whether and how nutritional status (*i.e*, fasted *vs* fed fish with or without dietary carbohydrates) affected global epigenome modifications by targeting DNA methylation and histone marks previously reported to be affected in metabolic diseases linked to disturbance of glucose metabolism (permissive H3K4me3, H3K9ac and H3K36me3, and repressive H3K9me3). As changes in the global epigenome induced by environmental modifications may have a potential epigenetic impact at the gene regulation level, we then examined whether dietary carbohydrates could affect the epigenetic landscape at duplicated gluconeogenic gene loci previously reported to display mRNA level changes in trout fed a high carbohydrate diet (*i.e, pck1, fbp1b1, fbp1b2, g6pca, g6pcb1a, g6pcb1b, g6pcb2a, g6pcb2b*^9^, and not *fbp1a* and *pck2* the mRNA levels of which remained stable). This last step would also provide new information to improve understanding of how duplicated genes encoding the same enzyme, but which are regulated differently by dietary carbohydrates, are regulated.

## Results

The fish used for the following analyses were fasted for four days and refed with either the NoCHO or the HighCHO diet and sampled 6 h after the last meal. Blood glucose levels to verify the hyperglycaemic phenotype in trout fed the HighCHO diet and expression patterns of duplicated gluconeogenic genes have already been published by Marandel *et al.*^9^.

### Analysis of global hepatic epigenome

**Figure 1:**
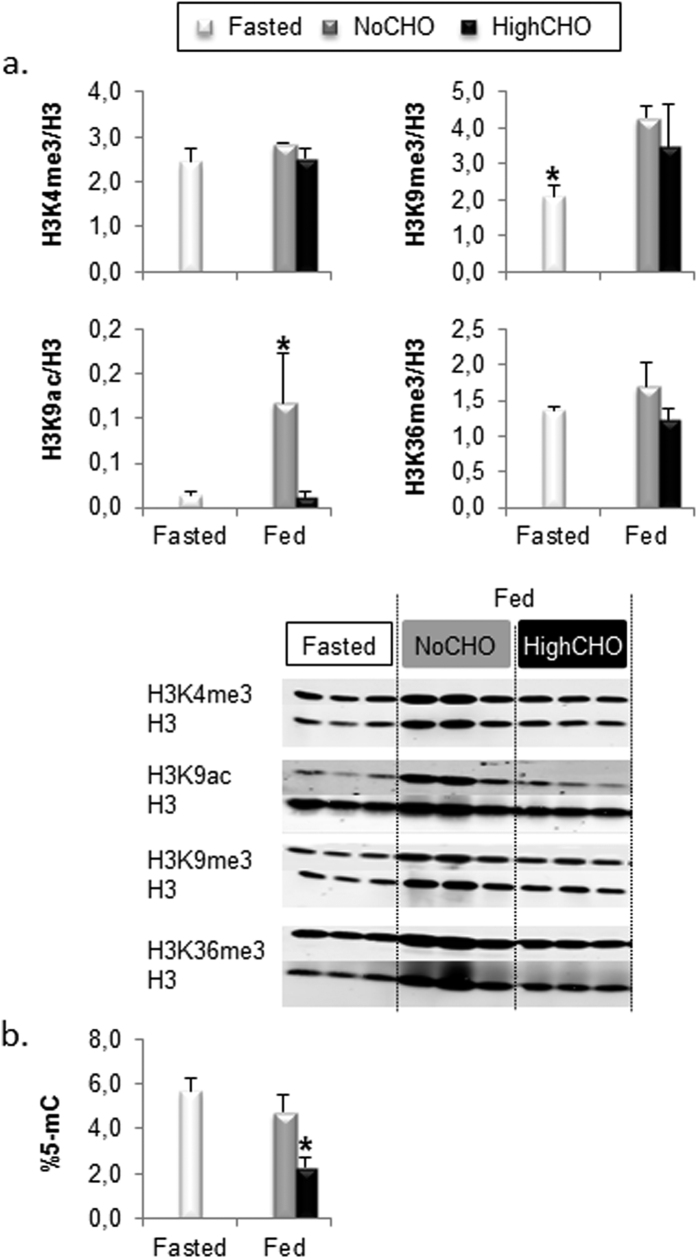
Global epigenetic modification in fasted trout (white bars), and those fed the NoCHO (grey bars) or the HighCHO (black bars) diet. Analyses of global histone modifications and representative blot (**A**), global DNA methylation (**B**). Data are expressed as mean ± SD, stars indicate significant differences between conditions (p < 0.01).

Global DNA methylation, H3K4me3, H3K9me3, H3K9ac and H3K36me3 levels were first assessed in fasted and fed trout (Fig. 1). Our analyses showed that the levels of H3K9ac (Fig. 1a) and the global DNA methylation (Fig. 1b) were affected in fed trout but in different ways. Indeed, trout fed the NoCHO diet displayed higher global enrichment in H3K9ac compared to fasted trout or those fed the HighCHO diet (Fig. 1a). However, DNA was hypomethylated in trout fed the HighCHO diet compared to fasted trout and fish fed the NoCHO diet (Fig. 1b). Our results also showed that fed trout displayed higher global enrichment in H3K9me3 compared to fasted fish whatever the composition of the diet (Fig. 1a). Finally, the global levels of H3K4me3 and H3K36me3 remained stable in fed trout whatever the nutritional status or the composition of the diet (Fig. 1a).

### Analyses of histone modifications at gluconeogenic gene loci

**Figure 2:**
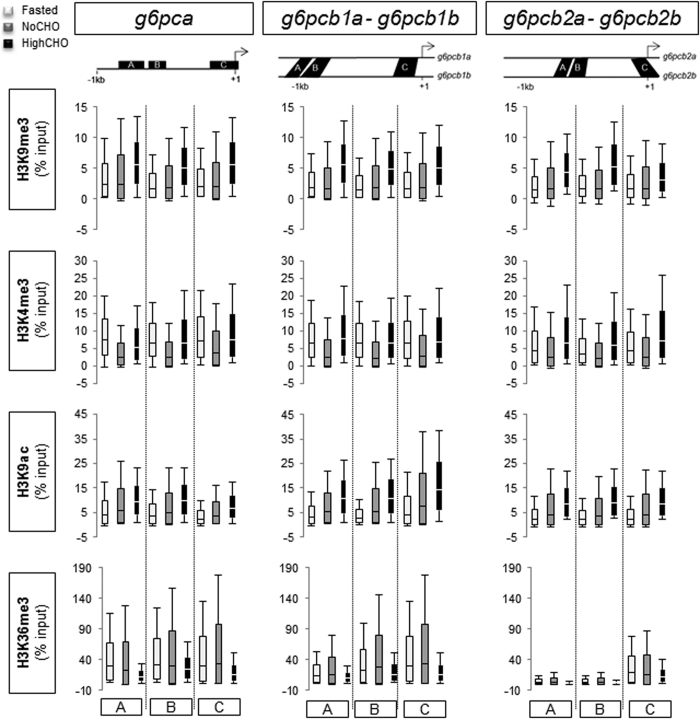
Histone modifications at *g6pc* gene loci in fasted trout (white), and those fed the NoCHO (grey) or the HighCHO (black) diet. The bottom and top of the box are mean-SD and mean + SD respectively, and the band inside the box is the mean. The ends of the whiskers represent the minimum and maximum of the data. Data represent the averages of three independent experiments. Analysed regions are identified with upper case letters and located with respect to transcription star site (identified by broken arrow).

**Figure 3:**
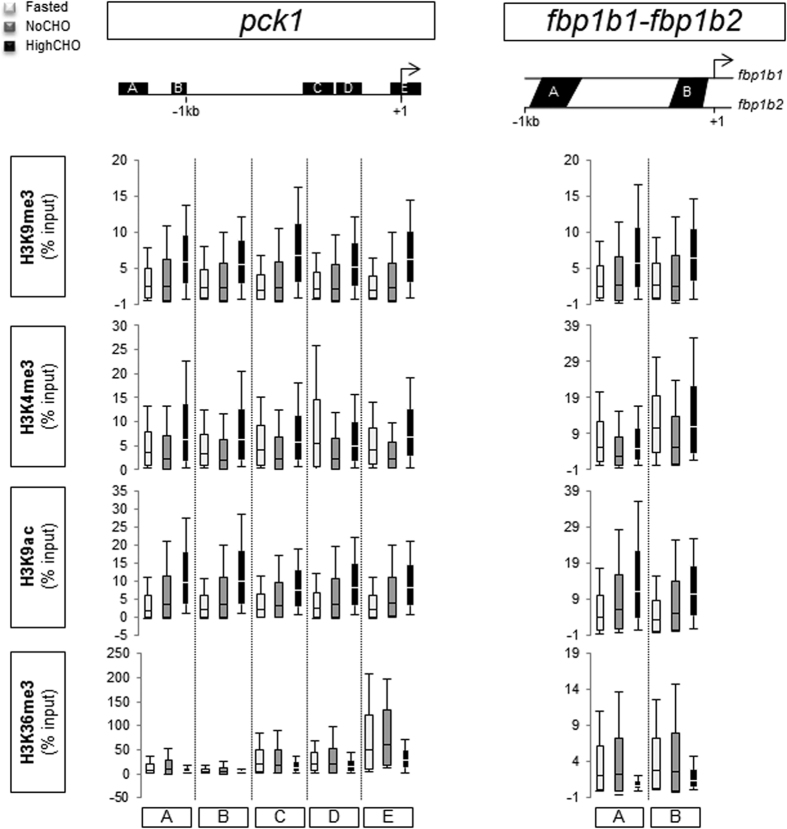
Histone modifications at *pck1* gene *and fbp1* gene loci in fasted trout (white), and those fed the NoCHO (grey) or the HighCHO (black) diet. The bottom and top of the box are mean-SD and mean + SD respectively, and the band inside the box is the mean. The ends of the whiskers represent the minimum and maximum of the data. Data represent the averages of three independent experiments. Analysed regions are identified with upper case letters and located with respect to transcription star site (identified by the broken arrow).

Classical ChIP -qPCR analyses were performed to profile the patterns of H3K4me3, H3K9me3, H3K9ac and H3K36me3 in relation to the nutritional status in trout along *g6pca*, *g6pcb1a/g6pcb1b*, *g6pcb2a/g6pcb2b* (Fig. 2), *pck1* and *fbp1b1/fbp1b2* (Fig. 3) 5′-upstream regions. No difference in enrichment level was evidenced whatever the nutritional status of the fish or the histone modification analysed. Results expressed as enrichment over the IgG mock (Supplementary Figure 1) revealed that H3K36me3 was more enriched around the TSS (Transcription Start Site) of the genes studied. Moreover, this mark globally displayed greater enrichment at gluconeogenic loci (except at *fbp1b1*/*fbp1b2* loci) in fasted animals and trout fed the NoCHO diet than in trout fed the HighCHO diet.

### Analysis of CpG methylation level at gluconeogenic gene loci

**Figure 4:**
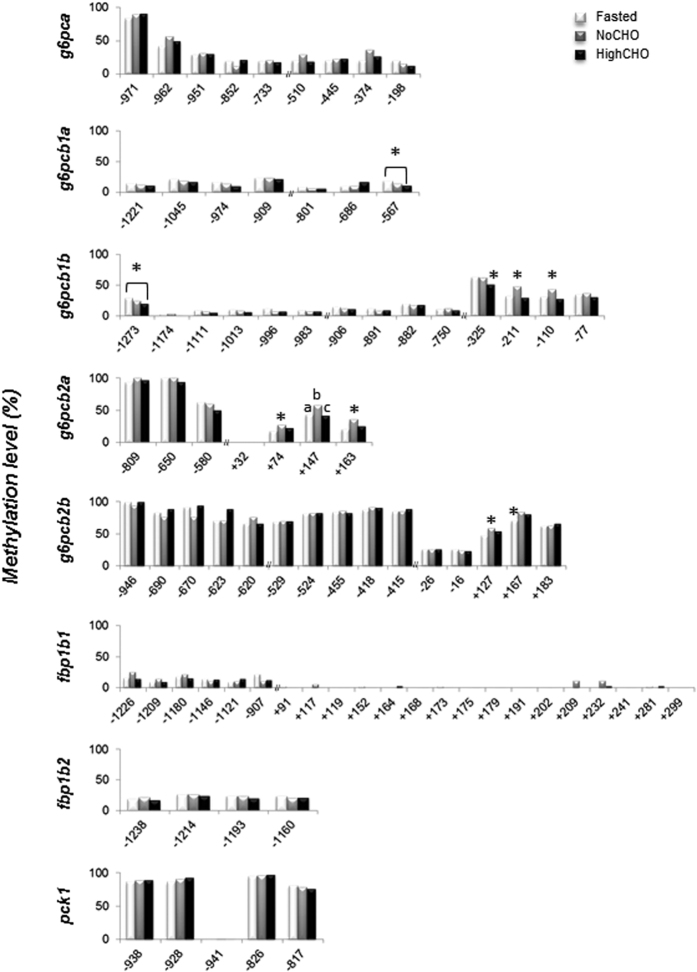
DNA methylation status of gluconeogenic genes in fasted trout (white bars), and those fed the NoCHO (grey bars) or the HighCHO (black bars) diet. Data are expressed as percentage of methylation at each CpG site. Positions of CpG sites are given in relation to the transcription start site. Different letters and stars indicate significant differences between conditions (p < 0.01).

Using targeted Next Generation Bisulfite Sequencing (t-NGBS), we analysed DNA methylation levels at specific CpG sites along the 5′-upstream region of gluconeogenic genes whose mRNA levels had previously been shown to be affected by nutritional status and/or dietary carbohydrates by Marandel *et al.*^9^,^10^,^11^ (i.e. *g6pc* paralogous genes, *fbp1b* ohnologs and *pck1*). DNA methylation levels of *g6pca*, *fbp1b1*, *fbp1b2* and *pck1*, remained stable whatever the nutritional condition (i.e., nutritional status and/or composition of the diet) or the CpG site analysed (Fig. 4). A significant decrease in the methylation level was observed at CpG sites −567 of *g6pcb1a* and −1273 and −325 of *g6pcb1b* in trout fed the HighCHO diet compared to fasted trout and trout fed the NoCHO diet. In contrast, an increase in DNA methylation level occurred in fed trout compared to fasted trout (at CpG site +167 of *g6pcb2b*) and in trout fed the NoCHO diet compared to fasted trout or trout fed the HighCHO diet (at CpG sites −211 and −110 of *g6pcb1b*; +74, +147 and +163 of *g6pcb2a*; and +127 of *g6pcb2b*).

## Discussion

Epigenetics refers to heritable changes in gene activity that are not caused by changes in the DNA sequence. Since one of their first definitions by Waddington^27^, epigenetic changes have been linked to environmental changes. By modifying the metabolic status of organisms and cells, nutrition is one of the recognised ways to induce epigenetic modifications and hence gene transcription activity changes. In this study we investigated histone modifications and DNA methylation changes, two epigenetic mechanisms. The results were analysed with regard to the nutritional status of fish, *i.e*. the feeding status (comparison of fasted and fed trout without dietary carbohydrates, the most common standard diet for trout in aquaculture) and then also according to the content of the diet in dietary carbohydrates (0% or 30%). We also examined the data in relation to the resulting hyperglycaemia observed in trout fed the HighCHO diet. Indeed, increase in blood glycaemia and its persistence can be considered an environmental factor and thus likely to induce epigenetic modifications.

We initially considered epigenetic modifications induced by the feeding status (i.e, between fasted and fed trout without dietary carbohydrates). The global analysis of the hepatic epigenome first revealed that H3K9me3 and H3K9ac levels were affected. Indeed, these two histone modifications were increased in trout fed the NoCHO diet compared to fasted trout, whereas H3K4me3, H3K36me3 and the DNA methylation level remained stable. To our knowledge, global epigenome changes in the livers of fasted and refed healthy animals have rarely been studied. These findings together suggested that the global hepatic epigenome was remodelled by the feeding status, and demonstrated that the global epigenome could be modified in the short term as fish were fed for only four days before sampling.

Secondly, histone modifications, which have an important role in the epigenetic regulation of chromatin dynamics and gene expression, are known to be affected by the feeding status (or by the levels of hormones involved in glucose homeostasis) in mammals and in diabetes at gluconeogenic gene loci^22^,^24^,^25^,^28^,^29^,^30^,^31^. In trout, we did not demonstrate any significant impact of the feeding status on the levels of H3K9me3, H3K4me3, H3K9ac and H3K36me3 along the 5′-upstream region of gluconeogenic genes. Several hypotheses could be proposed: 1) these histone modifications are not affected by such environmental factors at these precise loci in trout, 2) gluconeogenic genes in trout are not regulated by these histone modifications, 3) regions targeted for the analysis are not functional regulatory regions and 4) inter-individual variability erases environmental effects. With regard to this last hypothesis, we observed that the inherent variability monitored in each condition studied was not caused by the same individual from one histone modification to the other (data not shown). This result supported the fact that the variability detected was true biological variability and not due to technical impairment. Although we took care to make our sampling for chromatin extraction repeatable from one liver to another, this variability may reflect different composition of the liver tissue we extracted. Indeed, although hepatocytes accounted for 85% of the liver volume, the samples were not free from non-parenchymal cells^32^. As epigenetic mechanisms have an important role in cell differentiation, these two types of cell may display different epigenetic profiles. However, and remarkably, our findings revealed a H3K36me3 distribution profile that was different from those described in mammals and zebrafish. Indeed, in the latter species H3K36me3 was detected in gene bodies (exons), peaking towards the 3′ end^33^,^34^, whereas for most of the genes we studied (except for *fbp1ba/fbp1b2*) this modification was enriched around the TSS (Supplementary Figure 1). These findings suggest a potentially different role for this mark in trout at these loci or in general.

Finally, DNA methylation at specific gluconeogenic gene loci has previously been shown to be involved in the regulation of their expression mainly in cancer^35^,^36^,^37^ and also to be affected by *in utero* nutrition in mammals^22^,^38^. Our results showed that DNA methylation remained stable throughout the 5′-upstream region of *g6pca*, *fbp1b1*, *fbp1b2* and *pck1* whatever the feeding status of the animal. On the other hand, the methylation level was affected at specific CpG sites along *g6pcb1b*, *g6pcb2a* and *g6pcb2b* by the feeding status (*g6pcb1b*: sites −211 and −110, *g6pcb2a*: sites +74, +147, +163, and *g6pcb2b*: sites +127 and +167), all of them displaying higher methylation levels in fed trout than in fasted trout. These dynamic changes in DNA methylation at specific loci could not be correlated with changes in mRNA level described on the same samples by Marandel *et al.*^9^. Indeed, DNA methylation is generally (but not always) associated with gene repression^39^ but here all the genes mentioned above were non- (for *g6pcb1b*) or up-regulated (for *g6pcb2a* and *g6pcb2b*) in trout fed the NoCHO diet compared to fasted trout^9^.These findings suggest that DNA methylation did not directly regulate the gene under consideration in relation to the nutritional status but might interact with other regulatory mechanisms. It is noteworthy, however, that our analysis at specific loci also showed that overall *g6pcb2* ohnologs displayed higher DNA methylation levels than *g6pb1* ohnologs, and that *g6pca* was at an intermediate level between both pairs of ohnologs. This strongly confirms that these genes suffered a sub- or neo-functionalisation after the Ss4R at the epigenetic regulatory level which may have led to or participated in the previously described^9^,^10^,^11^ gene expression divergence. Indeed, several studies have recently demonstrated that sequence divergence and DNA methylation divergence of duplicated genes are initially combined, and that epigenetic modifications are important facilitators of duplicated gene evolution after a whole genome duplication event^40^,^41^,^42^.

These findings together showed for the first time that feeding status in trout, *i.e.* fasted vs fed state, affected the hepatic epigenetic landscape at the global level as well as at the level of some gluconeogenic genes even in the short term (only 4 days of starvation and feeding).

We also looked at the effects of dietary carbohydrates on the hepatic epigenetic landscape. Our results demonstrated that global DNA methylation and H3K9ac levels decreased in trout fed the HighCHO diet compared to trout fed the NoCHO diet, suggesting that dietary carbohydrates may directly or indirectly affect these two epigenetic marks. Hypomethylation of DNA and hypoacetylation of H3K9 have already been fully documented in metabolic diseases, particularly in diabetes (for DNA methylation^16^,^43^, and for H3K9ac^44^,^45^) and have often been linked to the hyperglycaemic state. This phenotype was also observed in a hyperglycaemia-induced model in zebrafish^21^,^46^ and under oxidative stress *in vitro*^47^. Although global hepatic H3K9 hypoacetylation remains to be investigated and understood, this modification seemed nevertheless closely linked to the hyperglycaemic state and to the metabolic memory phenomenon associated with the progression of diabetic complications^48^. Only the global DNA methylation profile has previously been investigated in trout fed diets containing 12% or 22% of dietary carbohydrates^26^. The latter study highlighted global hypomethylation of DNA in trout fed the 22% carbohydrate diet compared to trout fed the 12% carbohydrate diet, a result which is in accordance with our results. In addition, such global DNA hypomethylation occurred in trout fed the 22% carbohydrate diet with which hyperglycaemia was suppressed by methionine restriction. In diabetes and in a zebrafish hyperglycaemia-induced model it was proposed that oxidative stress induced by acute hyperglycaemia impaired genomic DNA methylation through the activation of the Tet-dependent iterative oxidation pathway^46^,^49^. This suggests that DNA hypomethylation could be induced by other mechanisms in trout or might not be due to the induced hyperglycaemia. It can be hypothesised that the increase in carbohydrate content alone or the decrease in protein content in the HighCHO diet might have been responsible for the decrease in DNA methylation. Indeed, in order to increase the carbohydrate content in our experimental HighCHO diet while keeping the same energy level as in the NoCHO diet, we changed on the carbohydrate/protein ratio, thus decreasing protein content from 61% to 40% 9. Protein restriction has been shown to modulate global and target gene DNA methylation levels^50^. Unexpectedly, we did not observe any changes in global H3K9me3 or H3K9me3 levels in trout fed the HighCHO diet. Actually, a decrease in global H3K9me3 level and an increase in H3K4me3 level have been reported in several studies related to diabetes and hyperglycaemia^20^,^51^. Our findings together suggested that the global hepatic epigenome was remodelled by the dietary carbohydrates, mimicking a phenotype previously described in diabetes at least for DNA methylation and H3K9ac. However, the causes of these epigenetic modifications (hyperglycaemia, protein or carbohydrate content in the diet) and the mechanism by which they were initiated remains to be elucidated.

With regard to histone modifications at gluconeogenic gene loci, as for the feeding status comparison, no significant impact of dietary carbohydrates on the levels of H3K9me3, H3K4me3, H3K9ac or H3K36me3 were demonstrated along the 5′-upstream region of gluconeogenic genes.

Finally, dynamic changes in DNA methylation at specific CpG sites were monitored at *g6pcb1a, g6pcb1b, g6pcb2a* and *g6pcb2b* loci in trout fed the HighCHO diet and compared to trout fed without dietary carbohydrates (*g6pcb1a*: site -567, *g6pcb1b*: sites −1273, −325, −211 and −110, *g6pcb2a*: sites +74, +147 and +163, and *g6pcb2b*: site +127). These findings demonstrated for the first time nutrient-induced promoter-specific methylation at *g6pc* loci in trout. In addition, when CpG methylation was affected in trout fed the HighCHO diet it was also in favour of a hypomethylation compared to trout fed the NoCHO diet and interestingly displayed the same level of methylation as in fasted trout. This pattern reflected what happened at the global epigenome level at these specific loci (see above). As explained above, DNA methylation acts in general to repress gene transcription. *g6pcb2* ohnologs have previously been shown to be up-regulated in trout fed the HighCHO diet and this was believed to contribute to the glucose intolerant phenotype^9^ in these fish. Hypomethylation of certain CpG sites at these two loci may thus be a potential contributor to the glucose intolerant phenotype *via* the de-repression of *g6pcb2* ohnologs. Functional analysis of affected CpG sites must now be tested to explore the relationship between their methylation levels and the expression levels of genes.

Taken together, our findings showed for the first time that changing dietary carbohydrate content in the trout diet has an effect on DNA methylation level at specific gluconeogenic gene loci and may be involved in the regulation of *g6pcb2* ohnologs previously believed to contribute to the initialisation of the glucose intolerant phenotype^9^ by their atypical up-regulation in trout fed the HighCHO diet.

## Conclusions

We reported here for the first time remodelling of the hepatic epigenetic landscape by nutritional status and dietary carbohydrate content of the trout diet both at global and target gene levels. We demonstrated that global hypomethylation occurred in trout fed the HighCHO diet which in particular mimicked a phenotype described in diabetes. DNA hypomethylation can lead to dramatic consequences such as induction of expression of oncogenes or miRNA^52^ and genomic instability^53^. The cause of this epigenetic phenotype must therefore now be clarified (i.e., decrease in protein content or increase in carbohydrate content in the diet or hyperglycaemia alone) as well as the mechanisms underlying its setting up in order to improve understanding of its impact on the glucose intolerant phenotype and its persistence in carnivorous teleosts.

## Methods

### Ethical issues and approval

Investigations were conducted according to the guiding principles for the use and care of laboratory animals and in compliance with French and European regulations on animal welfare (Décret 2001–464, 29 May 2001 and Directive 2010/63/EU, respectively). This protocol and the project as a whole were approved by the French National Consultative Ethics Committee.

### Diets and experimental design

Data published in this manuscript were obtained from the analysis of biological material used in an article previously published by Marandel *et al.*^9^. Juvenile rainbow trout (~70 g body mass) were reared at 17 °C in the INRA experimental facilities at Saint-Pée-sur-Nivelle, France. The first sampling was performed after four days of total starvation. Fish were then fed with either the NoCHO (containing no carbohydrate) or the HighCHO diet (containing 30% carbohydrates) twice a day at 2.5% live weight for four days and sampled 6h after the last meal in order to monitor the expected hyperglycaemic phenotype (around 0.8 g.L^−1^ for fasted trout and those fed the NoCHO diet and around 1.8 g.L^−1^ for trout fed the HighCHO diet, data previously published by Marandel *et al.*^9^). Diet compositions are provided as a reminder of Marandel *et al.*^9^ in Supplementary Table 1.

Gut content of each fish was systematically checked to confirm that the fish sampled had effectively consumed the diet. The livers were dissected and immediately frozen in liquid nitrogen, and stored at −80C until further analysis.

### Analysis of global DNA methylation

Livers of three fish per condition were analyzed. DNA extraction was performed on 10 mg of tissue as previously described^54^ without modification. DNA was quantified by NanoDrop (Thermo Fisher, USA) and the quality was verified on 1% agarose gel. The overall value of DNA methylation level (5-mC) was assessed using the MethylFlash Methylated DNA Quantification Kit (Epigentek, USA). Each analysis was performed in duplicate using 5ng of DNA, according to the manufacturer’s instructions.

### Analysis of global histones modifications

The livers of three fish per condition were analysed (same samples as for global DNA methylation analysis). One hundred mg of tissue was homogenized using Precellys24 (Bertin Technologies, Montigny-le-Bretonneux, France) in 2 ml tubes containing 1 ml of TEB (1X PBS, 0.5% Triton-100X, 5mM NaBu and 1X protease inhibitor from Roche (Cat. No. 04 693 116 001)) and four 2.8 millimeter ceramic beads, 2 × 10 s–15 s off at 5,000 rpm. Samples were then left to stand at 4 °C for 20 min before centrifugation at 2,000 rpm and 4 °C for 10 min. Pellets were then resuspended in 0.5 N HCl containing 10% glycerol (between 100 μl and 1,200 μl depending on the size of the pellet) and incubated on ice for 30 min (with vortexing every 10 min). Samples were centrifuged at 12,000 rpm at 4 °C for 5 min. Three volumes of iced acetone were added to the supernatant and precipitation was performed at 20°C overnight. Samples were centrifuged at 12,000 rpm at 4 °C for 5 min and iced cold acetone was used to wash the pellets 3 times. Pellets were resuspended in distilled water at 60 °C for 1h. Total protein concentration was determined using the Bio-Rad protein assay kit (Bio-Rad Laboratories, Germany). Lysates (5 μg of total protein) were subjected to SDS-PAGE and western blotting using the appropriate antibody on 15% gel (40% acrylamide, 2% bis-acrylamide, 1.5 M Tris-HCl pH 8.8, 10% SDS) for 40 min at 100 V and then 56 min at 150 V.

### Antibodies

The same antibodies were used for both western blotting and ChIP analysis. Anti-IgG (C15410206), Anti-H3K9me3 (C15410056), anti-H3K4me3 (C15410003), anti-H3K36me3 (C15410192) and anti-H3K9ac (C15410177) were purchased from Diagenode (Belgium) and anti-H3 (ab1791) from Abcam (UK).

### Analysis of target gene DNA methylation by targeted next-generation bisulfite sequencing

DNA extracted from the three livers per condition for global DNA analysis was used for gene-specific analysis of DNA methylation. Bisulfite conversion was performed using EZ DNA Methylation-Gold™ Kit (Zymo Research, D5005, USA) on each DNA sample according to the manufacturer’s instructions. PCR primers (Table 1) for the target regions were designed with the MethPrimer software (http://www.urogene.org/cgi-bin/methprimer/methprimer.cgi)^55^. To prevent any PCR skewing, three independent PCR amplifications were carried out using each bisulfite-converted DNA as template. Advantage® 2 Polymerase Mix (Clontech Laboratories, Inc., 639206, USA) was used for PCR amplification. The PCR conditions were 94 °C for 2 min and 40 cycles at 94 °C for 25 s, Tm for 1 min (Tm are given in Table 1 for each primers set) and 72 °C for 2 min, followed by 7 min at 72 °C. For each condition, all 9 PCR products obtained from the 3 original livers were pooled. Libraries were generated using the KAPA Library Preparation Kit (Kapa Biosystems, Wilmington, USA) at EpigenDx (Hopkinton, USA). Sequencing was performed at EpigenDx (Hopkinton, USA) on Ion Torrent PGM™ using a Ion 314™ Chip Kit v2. The NGS QC Toolkit v2.3.3 56 was used to trim data removing part of the sequences with a quality score lower than 18 followed by a removal of reads smaller than 35 nucleotides using Bowtie 2 57 using gene sequences *in silico* bisulfite converted as a reference. Alignment BAM files were then sorted by target and condition using BAMtools^58^ split function. Sorted reads were analyzed in BiQ Analyzer HT^59^ setting parameters at 100% of the read length, 90% sequence identity, bisulfite conversion efficiency ≥98% and lower cutoff at 30 reads per CpG site analyzed. The methylation level of each sampled cytosine was estimated as the number of reads reporting a C, divided by the total number of reads reporting a C or T. Positions of CpG sites were determined from the transcription start site (TSS, Fig. 4). Data were analyzed by a binomial global linear model followed by a Tukey test as a post-hoc analysis using the R Commander package in R (v.3.1.0)^60^.

### Analysis of modifications of histones at target gene loci by chromatin immunoprecipitation

The three livers previously used for DNA methylation analysis were investigated to monitor histone modifications at target gene loci. About 50 mg of frozen tissue was ground into small pieces in liquid nitrogen. Cross-linking was performed in 7.5 ml PBS containing 1% methanol-free formaldehyde for 10 min at room temperature (RT) on a rotating wheel. The cross-linking reaction was quenched by adding 0.12 M of glycine 5 min at RT. Samples were washed twice in 8 ml PBS and centrifuged for 10 min at 4 °C and 2000 g. Pellets were resuspended in 1 ml iced-cold PBS. Samples were homogenised using Precellys® 24 (Bertin Technologies, Montigny-le-Bretonneux, France) fitted with Cryolys® in 2 mL tubes containing six zirconium beads (2.8 millimiter), for 2 × 10 seconds, separated by 1min off, at 5,000 rpm. Homogenized samples were centrifuged for 10 min at 4 °C and 1300 g. Pellets were resuspended in 10ml classical lysis buffer (85 mM KCl, 5 mM PIPES pH8, 0.5% Igepas/NP40, 1X protease inhibitor cocktail (PI, P8340, Sigma-Aldrich Co) and 20mM NaBu) for 15 min at 4 °C on a rotating wheel and then centrifuged for 5 min at 4 °C and 1300 g. Extracted chromatin was resuspended in 130 μl of shearing buffer (10 mM EDTA, 50 mM Tris-HCl pH8, 1% SDS, 1X PI, 20 mM NaBu), left to stand on ice for 10 min and transferred to 1.5 ml Bioruptor® Plus TPX microtubes (Diagenode, Belgium). Shearing was performed in Bioruptor® Plus (Diagenode, Belgium): 7 cycles, 30 sec ON-30 sec OFF, High power. Sheared chromatin was centrifuged for 10 min at 4 °C and 14000 g and 10 μl was used to check the efficiency of the shearing (smear size expected between 150 bp and 500 bp). The remaining chromatin was diluted at 1:8 in immunoprecipitation (IP) buffer (0.5 mM EDTA, 1% Triton, 0.1% SDS, 1 mM EDTA, 10 mM Tris-HCl pH8, 140 mM NaCl, 0.1% Na-deoxycholate, 1X PI, 20 mM NaBu). Ten μl of Dynabeads® Protein A (Life Technologies, USA) were coated with 3 μg of antibody (including IgG to evaluate the background) diluted in 90 μl IP buffer for 2 h at 4 °C. Coated beads were resuspended in 100 μl of diluted chromatin overnight at 4°C. Beads were washed four times in IP buffer, and once in 100 μl TE buffer (10 mM EDTA, 10 mM Tris-HCl pH8). Immunoprecipitated chromatin was finally eluted in 150 μl of ChIP elution buffer (5 mM EDTA, 20 mM Tris-HCl pH 7.5, 1% SDS, 50 mM NaCl, 0.063μg proteinase K), decrosslinked for 2 h at 68 °C, 1300 rpm, and resuspended in a 900 μl final volume of ChIP elution buffer. Ten μl of diluted chromatin was also decrosslinked to assess input. DNA from immunoprecipitated chromatin and input were isolated by classical phenol-chloroform extraction and resuspended in a 15 μl final volume of DNAse-free water. The Roche Lightcycler 480 system was used for qPCR (Roche Diagnostics, Neuilly-sur-Seine, France). The primer sequences used are listed in Table 2. Primers were tested on input DNA from previous ChIP assays for validation. Amplified products were systematically sequenced. When a pair of ohnologous genes was analysed (for instance *g6pcb2a* and *g6pcb2b*), the same primer set amplified both genes as we were not able to design specific primers because of the high percentage of identity between the ohnologous sequences. The assays were performed using a reaction mix of 6 μl per sample, each of which contained 2 μl of diluted DNA template, 0.3 μl of each primer (5 μM), 3 μl Light Cycler 480 SYBR® Green I Master mix and 0.7 μl DNAse/RNAse free water (5 Prime GmbH, Hamburg, Germany). The PCR protocol was initiated at 95 °C for 10 min for initial denaturation of the DNA and hot-start Taq-polymerase activation, followed by 45 cycles of a two-step amplification programme (15 s at 95 °C; 40 s at 60 °C). Each PCR assay was performed in duplicate. Results were expressed as % input and fold enrichment over IgG (see Supplementary Figure 1). Normality of distributions was assessed using the Shapiro-Wilk test. Data were then analysed by a Kruskal-Wallis nonparametric test following by a Tukey test as a post hoc analysis. Data were analysed with the R Commander package in R (v.3.1.0)^60^.

## Additional Information

**How to cite this article**: Marandel, L. *et al.* Remodelling of the hepatic epigenetic landscape of glucose-intolerant rainbow trout (*Oncorhynchus mykiss*) by nutritional status and dietary carbohydrates. *Sci. Rep.* **6**, 32187; doi: 10.1038/srep32187 (2016).

## Supplementary Material

Supplementary Information

## Figures and Tables

**Table 1:** Primers used for gene-specific DNA methylation analysis.

Gene	Location (from TSS)	Primers (5′-3′)	Tm
*g6pca*	−1133/−681	F: GTTTTTGTAAGATTATGTAATG	55.8
		R: TCATTTACTATTTCCTTCCC	
	−552/−158	F: TGGATTATTTGAAGTGTTTTTGTTATAATT	55.8
		R: AATTAACCCTACCCCACCTTATAAA	
*g6pcb1a*	−1246/−855	F: AATGAAAATTGATTGATTTA	55.8
		R: AAATCTAACCAAAATCCTA	
	−866/−530	F: TTTTGTGGGTAAAATATTTGATTG	55.8
		R: TCAATTTTCATTAAACTACTTAAAATATCC	
*g6pcb1b*	−1295/−941	F: ATTTAGATTAGTAATTTAGGG	55.8
		R: AATCAATCAATAAATCAATCA	
	−962/−620	F: TTGATTGATTTATTGATTGATTTTTTT	60.5
		R: TCAATATCCTATAACTAACCCACCTATC	
	−359/−9	F: ATTGGTTAAAAAGGGGGTTTAGTAA	60.5
		R: ACAAAAAAAACCATACAAACAAATACA	
*g6pcb2a*	−963/−527	F: GTTATTTAGAATTTAAAAGG	55.8
		R: CTCAAAATAAATAAATACCA	
	−52/+208	F: TGGTAGTGGTGATAGGTGGATATT	55.8
		R: TCCATAACTACTCTCTAATTCACTATATCT	
*g6pcb2b*	−972/−568	F: GTTATTTAGAATTTAAAAGG	55.8
		R: CTCAAAATAAATAAATACCA	
	−562/−385	F: TAATAGGGTAGGGAGTGATAATTGG	55.8
		R: CATCAAACATATAAAAACCACATACTTAAC	
	−54/+211	F: TTTATATAGGGTATAAAAGGGATAGTAG	60.5
		R: ATTCACTATATCTAACAATCACACTAAC	
*fbp1b1*	−1360/−916	F: TGAAGGTTTTGTTAATAATAGAAAAA	55.8
		R: AAATCATACATAAATCAATCCAATATATTA	
	−77/+280	F: TGAAAGGTTAATTGTGATTGGTTTA	55
		R: AACAATCCCAACTTTTCTAACAAC	
*fbp1b2*	−1339/−1052	F: GAATTTGGTTTTTGATTTTTGT	60.5
		R: TTTTAACCAAATCCCTATAAACC	
*pck1*	−1007/−579	F: TGATTGTGTTTTATTTGTTGTT	60
		R: AACAAACTAAACCAAATTTCTTT	

**Table 2:** Primers used for gene-specific histone modification analyses (all were used at 60 °C).

Gene(s)	Location from TSS	Primers (5′–3′)
*g6pca*	−809/−681 (A)	F: TCAGGAGATGCTGAGAAGATAAC
		R: TCATTTGCTATTTCCTTCCCAGA
	−626/−518 (B)	F: GTTCCATTCGTTTCACATGCC
		R: GTGAAGATTGTAGCAAGGACACT
	−215/+3 (C)	F: GGCCTCCAAATCACCAAGTC
		R:CATGCAGTCTGTTGTTCCCA
*g6pcb1a/g6pcb1b*	−959/−1047 (A- *g6pcb1a*)	F: TCCGCCACTGAGCCTACA
	−1158/−1063 (A- *g6pc1b*)	R: GTTTCAGGGGCTAGCGTATC
	−901/−789 (B- *g6pcb1a*)	F: GGTGAAGATTGTAGCAAGGACAC
	−1005/−879 (B- *g6pcb1b*)	R: TGAGTTCCATTCGTTTCACATG
	−70/−183 (C- *g6pcb1a*)	F: CCTTGCTTGGCTTGTTTTGC
	−195/−80 (C- *g6pcb1b*)	R: CACCTGAAATGGAGCCAAAAA
*g6pcb2a/g6pcb2b*	−620/−528 (A- *g6pcb2a*)	F: TGTTCAACTCCACTACCCCA
	−660/−568 (A- *g6pcb2b*)	R: TCAGAGTGAGTAGATGCCAGA
	−517/−403 (B- *g6pcb2a*)	F: GGGCAGGGAGCGATAACTGG
	−557/−462 (B- *g6pcb2b*)	R: TATTGTTCCTCCCACCAGC
	−108/−13 (C- *g6pcb2a*)	F: TTCAAAGATCAGGCGTGGTG
	−87/+9 (C- *g6pcb2b*)	R: TGCTACTTGTCTGTCCAGTG
*fbp1b1-fbp1b2*	−929/−732 (A- *fbp1b1*)	F: GAGCAACAAACAGAACCCAATG
	−988/−807 (A- *fbp1b2*)	R: AGCACATTGGTTTAACAGCCT
	−206/−35 (B- *fbp1b1*)	F: ATTGCTTACCAGTCCTTTCAGAT
	−246/−46 (B- *fbp1b2*)	R: AACCAATCACAGTTGGCCTTTCA
*pck1*	−1305/−1184 (A)	F: TGGCCAAGTCAAAGTCCAGA
		R: CCCATTCCTCCTTGCAAAACA
	−1076/−965 (B)	F: GCTGAATAATTTTGCACGCCC
		R: AGAATCAACAACAAGTGGGACA
	−378/−249 (C)	F: TCAAGGATCGGCACATTCCT
		R: AGTGATTCAACAGTTTCGCTCT
	−310/−166 (D)	F: GCCTCCAAAATGTGCCAATAG
		R: CAACTGAGCATCTTGTTCTTTCA
	−107/+44 (E)	F: CAGAGTTTTCCAAGAGCTGAACA
		R: GGGCTGTTCTTGAATTGTATCCA

Letters in brackets refer to the location of amplified regions identified in Figs 2 and 3.

## References

[b1] Bergot F. Specific problems posed by carbohydrate utilization in the rainbow trout. Ann Nutr Aliment 33, 247–257 (1979). 496176

[b2] Polakof S., Moon T. W., Aguirre P., Skiba-Cassy S. & Panserat S. Glucose homeostasis in rainbow trout fed a high-carbohydrate diet: metformin and insulin interact in a tissue-dependent manner. Am J Physiol Regul Integr Comp Physiol. 300, R166–R174, doi: 10.1152/ajpregu.00619.2010 (2011). 21068202

[b3] Skiba-Cassy S. *et al.* Apparent low ability of liver and muscle to adapt to variation of dietary carbohydrate:protein ratio in rainbow trout (Oncorhynchus mykiss). Br J Nutr. 109, 1359–1372, doi: 10.1017/s0007114512003352 (2013). 22951215

[b4] del sol Novoa M. *et al.* Glucagon and insulin response to dietary carbohydrate in rainbow trout (Oncorhynchus mykiss). Gen Comp Endocrinol. 139, 48–54, doi: 10.1016/j.ygcen.2004.07.005 (2004). 15474535

[b5] Krasnov A., Teerijoki H. & Molsa H. Rainbow trout (Onchorhynchus mykiss) hepatic glucose transporter. Biochim Biophys Acta 1520, 174–178 (2001). 11513960 10.1016/s0167-4781(01)00258-5

[b6] Panserat S. *et al.* Hepatic glucokinase is induced by dietary carbohydrates in rainbow trout, gilthead seabream, and common carp. Am J Physiol Regul Integr Comp Physiol. 278, R1164–R1170 (2000). 10801283 10.1152/ajpregu.2000.278.5.R1164

[b7] Polakof S., Panserat S., Soengas J. L. & Moon T. W. Glucose metabolism in fish: a review. J Comp Physiol B 182, 1015–1045, doi: 10.1007/s00360-012-0658-7 (2012). 22476584

[b8] Berthelot C. *et al.* The rainbow trout genome provides novel insights into evolution after whole-genome duplication in vertebrates. Nat Commun. 5, 3657, doi: 10.1038/ncomms4657 (2014). 24755649 PMC4071752

[b9] Marandel L., Seiliez I., Veron V., Skiba-Cassy S. & Panserat S. New insights into the nutritional regulation of gluconeogenesis in carnivorous rainbow trout (Oncorhynchus mykiss): a gene duplication trail. Physiol Genomics 47, 253–263, doi: 10.1152/physiolgenomics.00026.2015 (2015). 25901068

[b10] Marandel L., Veron V., Surget A., Plagnes-Juan E. & Panserat S. Glucose metabolism ontogenesis in rainbow trout (Oncorhynchus mykiss) in the light of the recently sequenced genome: new tools for intermediary metabolism programming. J Exp Biol. 219, 734–743, doi: 10.1242/jeb.134304 (2016). 26747908

[b11] Marandel L., Dai W., Panserat S. & Skiba-Cassy S. The five glucose-6-phosphatase paralogous genes are differentially regulated by insulin alone or combined with high level of amino acids and/or glucose in trout hepatocytes. Mol Biol Rep. 43, 207–211, doi: 10.1007/s11033-016-3962-6 (2016). 26896939

[b12] Jimenez-Chillaron J. C. *et al.* The role of nutrition on epigenetic modifications and their implications on health. Biochimie. 94, 2242–2263, doi: 10.1016/j.biochi.2012.06.012 (2012). 22771843

[b13] Ulrey C. L., Liu L., Andrews L. G. & Tollefsbol T. O. The impact of metabolism on DNA methylation. Hum Mol Genet 14 Spec No 1, R139–R147, doi: 10.1093/hmg/ddi100 (2005). 15809266

[b14] Lu C. & Thompson C. B. Metabolic regulation of epigenetics. Cell Metab. 16, 9–17, doi: 10.1016/j.cmet.2012.06.001 (2012). 22768835 PMC3392647

[b15] Katada S., Imhof A. & Sassone-Corsi P. Connecting threads: epigenetics and metabolism. Cell 148, 24–28, doi: 10.1016/j.cell.2012.01.001 (2012). 22265398

[b16] Williams K. T., Garrow T. A. & Schalinske K. L. Type I diabetes leads to tissue-specific DNA hypomethylation in male rats. J Nutr. 138, 2064–2069, doi: 10.3945/jn.108.094144 (2008). 18936199

[b17] de Mello V. D. *et al.* DNA methylation in obesity and type 2 diabetes. Ann Med. 46, 103–113, doi: 10.3109/07853890.2013.857259 (2014). 24779963

[b18] Tu P. L., Ma X., Zhang B., Duan Y., Ni H., Wang Z., Jiang H., Li P., Wu M. & Li R. M. Changes and significance of hepatic histone H3 epigenetic modification in type 2 diabetic mice. Journal of Jilin University Medicine Edition 41, 756–762 (2015).

[b19] Chiang E. P., Wang Y. C., Chen W. W. & Tang F. Y. Effects of insulin and glucose on cellular metabolic fluxes in homocysteine transsulfuration, remethylation, S-adenosylmethionine synthesis, and global deoxyribonucleic acid methylation. J Clin Endocrinol Metab 94, 1017–1025, doi: 10.1210/jc.2008-2038 (2009). 19088160

[b20] El-Osta A. *et al.* Transient high glucose causes persistent epigenetic changes and altered gene expression during subsequent normoglycemia. J Exp Med. 205, 2409–2417, doi: 10.1084/jem.20081188 (2008). 18809715 PMC2556800

[b21] Olsen A. S., Sarras M. P. Jr., Leontovich A. & Intine R. V. Heritable transmission of diabetic metabolic memory in zebrafish correlates with DNA hypomethylation and aberrant gene expression. Diabetes 61, 485–491, doi: 10.2337/db11-0588 (2012). 22228713 PMC3266410

[b22] Jia Y. *et al.* Maternal low-protein diet induces gender-dependent changes in epigenetic regulation of the glucose-6-phosphatase gene in newborn piglet liver. J Nutr. 142, 1659–1665, doi: 10.3945/jn.112.160341 (2012). 22833655

[b23] Burdge G. C. *et al.* Progressive, transgenerational changes in offspring phenotype and epigenotype following nutritional transition. Plos One 6, e28282, doi: 10.1371/journal.pone.0028282 (2011). 22140567 PMC3227644

[b24] Pan D. *et al.* The histone demethylase Jhdm1a regulates hepatic gluconeogenesis. Plos Genet 8, e1002761, doi: 10.1371/journal.pgen.1002761 (2012). 22719268 PMC3375226

[b25] Ravnskjaer K. *et al.* Glucagon regulates gluconeogenesis through KAT2B- and WDR5-mediated epigenetic effects. J Clin Invest 123, 4318–4328, doi: 10.1172/jci69035 (2013). 24051374 PMC3784539

[b26] Craig P. M. & Moon T. W. Methionine restriction affects the phenotypic and transcriptional response of rainbow trout (Oncorhynchus mykiss) to carbohydrate-enriched diets. Br J Nutr. 109, 402–412, doi: 10.1017/s0007114512001663 (2013). 22583536

[b27] Principles of Embryology. *Allen and Unwin Eds.*, *London* (1956).

[b28] Hall R. K., Wang X. L., George L., Koch S. R. & Granner D. K. Insulin represses phosphoenolpyruvate carboxykinase gene transcription by causing the rapid disruption of an active transcription complex: a potential epigenetic effect. Mol Endocrinol. 21, 550–563, doi: 10.1210/me.2006-0307 (2007). 17095578

[b29] Tsai W. W. *et al.* PRMT5 modulates the metabolic response to fasting signals. Proc Natl Acad Sci USA 110, 8870–8875, doi: 10.1073/pnas.1304602110 (2013). 23671120 PMC3670393

[b30] Strakovsky R. S., Zhang X., Zhou D. & Pan Y. X. Gestational high fat diet programs hepatic phosphoenolpyruvate carboxykinase gene expression and histone modification in neonatal offspring rats.J Physiol. 589, 2707–2717, doi: 10.1113/jphysiol.2010.203950 (2011). 21486814 PMC3112549

[b31] Lillycrop K. A. *et al.* Induction of altered epigenetic regulation of the hepatic glucocorticoid receptor in the offspring of rats fed a protein-restricted diet during pregnancy suggests that reduced DNA methyltransferase-1 expression is involved in impaired DNA methylation and changes in histone modifications. Br J Nutr. 97, 1064–1073, doi: 10.1017/s000711450769196x (2007). 17433129 PMC2211425

[b32] Hampton J. A., Lantz R. C. & Hinton D. E. Functional units in rainbow trout (Salmo gairdneri, Richardson) liver: III. Morphometric analysis of parenchyma, stroma, and component cell types. Am J Anat 185, 58–73, doi: 10.1002/aja.1001850107 (1989). 2782277

[b33] Hon G. C., Hawkins R. D. & Ren B. Predictive chromatin signatures in the mammalian genome. Hum Mol Genet 18, R195–R201, doi: 10.1093/hmg/ddp409 (2009). 19808796 PMC2912651

[b34] Vastenhouw N. L. *et al.* Chromatin signature of embryonic pluripotency is established during genome activation. Nature 464, 922–926, doi: 10.1038/nature08866 (2010). 20336069 PMC2874748

[b35] Bigl M., Jandrig B., Horn L. C. & Eschrich K. Aberrant methylation of human L- and M-fructose 1,6-bisphosphatase genes in cancer. Biochem Biophys Res Commun. 377, 720–724, doi: 10.1016/j.bbrc.2008.10.045 (2008). 18938139

[b36] Liu X. *et al.* Warburg effect revisited: an epigenetic link between glycolysis and gastric carcinogenesis. Oncogene 29, 442–450, doi: 10.1038/onc.2009.332 (2010). 19881551

[b37] Chen M. *et al.* Promoter hypermethylation mediated downregulation of FBP1 in human hepatocellular carcinoma and colon cancer. Plos One 6, e25564, doi: 10.1371/journal.pone.0025564 (2011). 22039417 PMC3198434

[b38] Nijland M. J. *et al.* Epigenetic modification of fetal baboon hepatic phosphoenolpyruvate carboxykinase following exposure to moderately reduced nutrient availability.J Physiol. 588, 1349–1359, doi: 10.1113/jphysiol.2009.184168 (2010). 20176628 PMC2872738

[b39] Bird A. DNA methylation patterns and epigenetic memory.Genes Dev. 16, 6–21, doi: 10.1101/gad.947102 (2002). 11782440

[b40] Wang H. *et al.* CG gene body DNA methylation changes and evolution of duplicated genes in cassava. Proc Natl Acad Sci USA 112, 13729–13734, doi: 10.1073/pnas.1519067112 (2015). 26483493 PMC4640745

[b41] Keller T. E. & Yi S. V. DNA methylation and evolution of duplicate genes. Proc Natl Acad Sci USA 111, 5932–5937, doi: 10.1073/pnas.1321420111 (2014). 24711408 PMC4000835

[b42] Mendizabal I., Keller T. E., Zeng J. & Yi S. V. Epigenetics and evolution. Integr Comp Biol. 54, 31–42, doi: 10.1093/icb/icu040 (2014). 24838745 PMC6394367

[b43] Sanchez I., Reynoso-Camacho R. & Salgado L. M. The diet-induced metabolic syndrome is accompanied by whole-genome epigenetic changes. Genes Nutr. 10, 471, doi: 10.1007/s12263-015-0471-5 (2015). 25998092 PMC4440867

[b44] Tu P. *et al.* Liver histone H3 methylation and acetylation may associate with type 2 diabetes development. J Physiol Biochem. 71, 89–98, doi: 10.1007/s13105-015-0385-0 (2015). 25666660

[b45] Cencioni C. *et al.* Epigenetic mechanisms of hyperglycemic memory. Int J Biochem Cell Biol. 51, 155–158, doi: 10.1016/j.biocel.2014.04.014 (2014). 24786298

[b46] Dhliwayo N., Sarras M. P. Jr., Luczkowski E., Mason S. M. & Intine R. V. Parp inhibition prevents ten-eleven translocase enzyme activation and hyperglycemia-induced DNA demethylation. Diabetes 63, 3069–3076, doi: 10.2337/db13-1916 (2014). 24722243 PMC4141369

[b47] Niu Y., DesMarais T. L., Tong Z., Yao Y. & Costa M. Oxidative stress alters global histone modification and DNA methylation. Free Radic Biol Med. 82, 22–28, doi: 10.1016/j.freeradbiomed.2015.01.028 (2015). 25656994 PMC4464695

[b48] Zhong Q. & Kowluru R. A. Role of histone acetylation in the development of diabetic retinopathy and the metabolic memory phenomenon. J Cell Biochem. 110, 1306–1313, doi: 10.1002/jcb.22644 (2010). 20564224 PMC2907436

[b49] Ding G. L. & Huang H. F. Role for tet in hyperglycemia-induced demethylation: a novel mechanism of diabetic metabolic memory. Diabetes 63, 2906–2908, doi: 10.2337/db14-0675 (2014). 25146472

[b50] Zhang N. Epigenetic modulation of DNA methylation by nutrition and its mechanisms in animals. Animal nutrition 1, 144–151 (2015). 10.1016/j.aninu.2015.09.002 PMC5945948 29767106

[b51] Siebel A. L., Fernandez A. Z. & El-Osta A. Glycemic memory associated epigenetic changes. Biochem Pharmacol. 80, 1853–1859, doi: 10.1016/j.bcp.2010.06.005 (2010). 20599797

[b52] Han L., Witmer P. D., Casey E., Valle D. & Sukumar S. DNA methylation regulates MicroRNA expression. Cancer Biol Ther. 6, 1284–1288 (2007). 17660710 10.4161/cbt.6.8.4486

[b53] Rodriguez J. *et al.* Chromosomal instability correlates with genome-wide DNA demethylation in human primary colorectal cancers. Cancer Res. 66, 8462–9468, doi: 10.1158/0008-5472.can-06-0293 (2006). 16951157

[b54] Marandel L., Labbe C., Bobe J. & Le Bail P. Y. Evolutionary history of c-myc in teleosts and characterization of the duplicated c-myca genes in goldfish embryos. Mol Reprod Dev. 79, 85–96, doi: 10.1002/mrd.22004 (2012). 22213278

[b55] Li L. C. & Dahiya R. MethPrimer: designing primers for methylation PCRs. Bioinformatics 18, 1427–1431 (2002). 12424112 10.1093/bioinformatics/18.11.1427

[b56] Patel R. K. & Jain M. NGS QC Toolkit: a toolkit for quality control of next generation sequencing data. Plos One 7, e30619, doi: 10.1371/journal.pone.0030619 (2012). 22312429 PMC3270013

[b57] Langmead B. & Salzberg S. L. Fast gapped-read alignment with Bowtie 2. Nat Methods 9, 357–359, doi: 10.1038/nmeth.1923 (2012). 22388286 PMC3322381

[b58] Barnett D. W., Garrison E. K., Quinlan A. R., Stromberg M. P. & Marth G. T. BamTools: a C++ API and toolkit for analyzing and managing BAM files. Bioinformatics 27, 1691–1692, doi: 10.1093/bioinformatics/btr174 (2011). 21493652 PMC3106182

[b59] Lutsik P. *et al.* BiQ Analyzer HT: locus-specific analysis of DNA methylation by high-throughput bisulfite sequencing. Nucleic Acids Res. 39, W551–W556, doi: 10.1093/nar/gkr312 (2011). 21565797 PMC3125748

[b60] Fox J. The R Commander: A basic statistics graphical user interface to R. Journal of Statistical Software 14, 1–42 (2005).

