# Supplementary material for: Remodelling of the hepatic epigenetic landscape of glucose-intolerant rainbow trout (Oncorhynchus mykiss) by nutritional status and dietary carbohydrates
Source: Sci Rep. 2016 Aug 26;6:32187. doi: 10.1038/srep32187 (PMC4999891; doi:10.1038/srep32187)
Supplement: Supplementary Information [file srep32187-s1.pdf]

Remodelling of the hepatic epigenetic landscape of glucose-intolerant rainbow trout (Oncorhynchus mykiss) by nutritional status and dietary carbohydrates

Lucie Marandel*1, Olivier Lepais2, Eva Arbenoits1, Vincent Véron1, Karine Dias1, Marie Zion1, Stéphane Panserat1

Supplementary Information

Supplementary Table 1. Formulation and proximate composition of the two experimental diets used (NoCHO and HighCHO diets) in this experiment.

	**Diets**	
	**NoCHO**	**HighCHO**
**Ingredients %**		
Fish meal[1]	90.85	55.9
Fish oil[2]	5.15	10.1
Starch[3]	0	30
Vitamin mix[4]	1	1
Mineral mix[5]	1	1
Alginate[6]	2	2
**Proximate composition**		
Dry matter (DM, % diet)	95.46	94.69
Crude protein (% DM)	61.30	39.08
Crude lipid (% DM)	18.86	17.70
Gross energy (kJ/g DM)	22.05	21.76
Ash (% DM)	17.73	11.38
Carbohydrates (% DM)	<1.0	28.4

[1] Sopropeche, Boulogne-sur-Mer, France
[2] fish oil; Sopropeche, Boulogne-sur-Mer, France.
[3] Gelatinized corn starch (Roquette, Lestrem, France)
[4] Supplied the following (kg^{-1} diet): DL-a tocopherol acetate 60 IU, sodium menadione bisulphate 5 mg, retinyl acetate 15000 IU, DLcholecalciferol 3000 IU, thiamin 15 mg, riboflavin 30 mg, pyridoxine 15 mg, vit. B_{12} 0.05 mg, nicotinic acid 175 mg, folic acid 500 mg, inositol 1000 mg, biotin 2.5 mg, calcium panthotenate 50 mg, choline chloride 2000 mg.
[5] Supplied the following (kg^{-1} diet): calcium carbonate (40% Ca) 2.15 g, magnesium oxide (60% Mg) 1.24 g, ferric citrate 0.2 g, potassium iodide (75% I) 0.4 mg, zinc sulphate (36% Zn) 0.4 g, copper sulphate (25% Cu) 0.3 g, manganese sulphate (33% Mn) 0.3 g, dibasic calcium phosphate (20% Ca, 18% P) 5 g, cobalt sulphate 2 mg, sodium selenite (30% Se) 3 mg, potassium chloride 0.9 g, Sodium chloride 0.4 g.
[6] Louis François, Marne-la-Vallée, France

Supplemental Figure 1

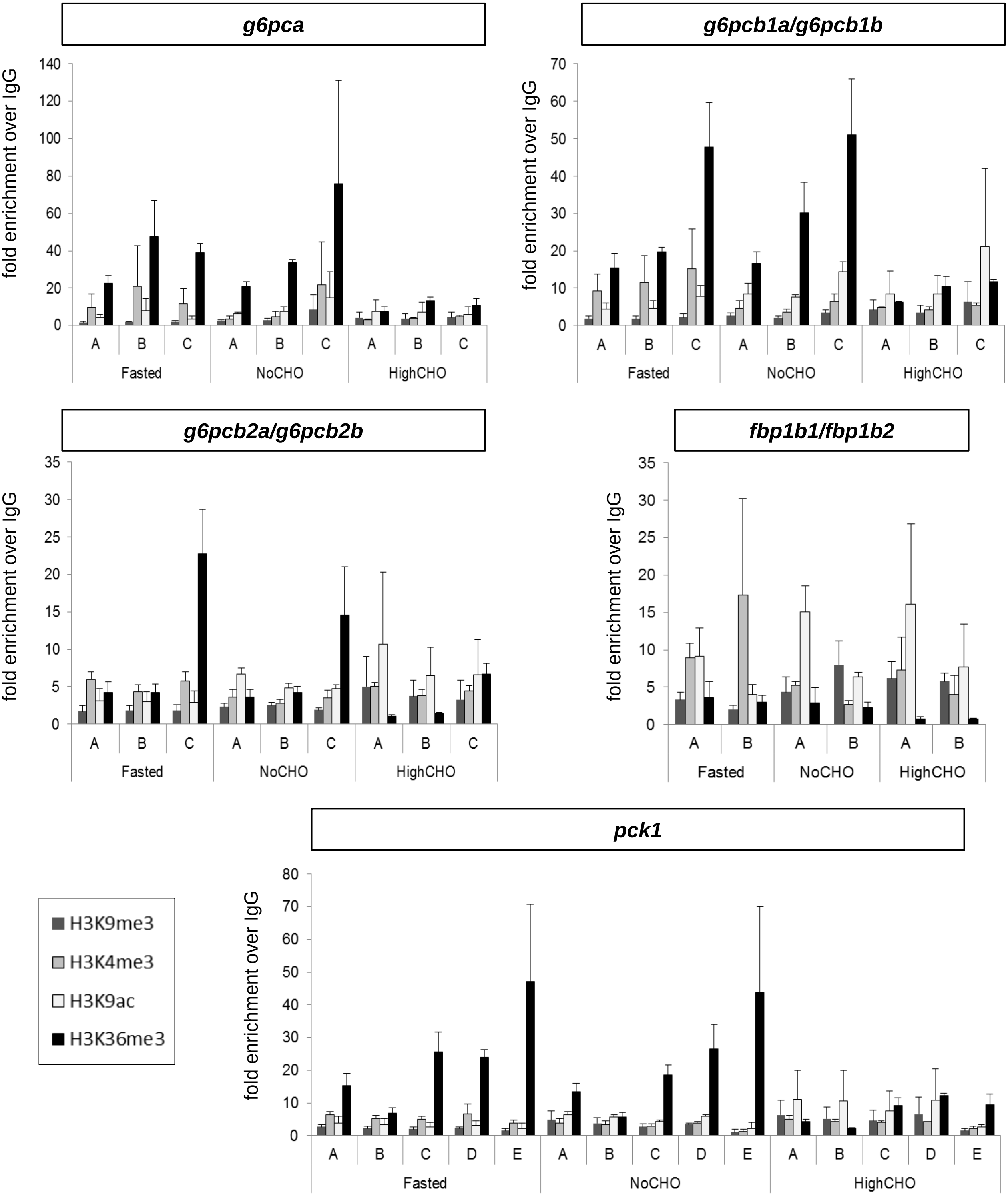

Supplementary Figure 1 – Histone modifications at gluconeogenic gene loci in fasted trout (white), and those fed the NoCHO (grey) or the HighCHO (black) diet expressed as fold enrichment over IgG. Data are expressed as mean ± SD and represent the averages of three independent experiments.
